# Supplementary material for: Impact of the Vaginal and Endometrial Microbiome Pattern on Assisted Reproduction Outcomes
Source: J Clin Med. 2021 Sep 8;10(18):4063. doi: 10.3390/jcm10184063 (PMC8465380; doi:10.3390/jcm10184063)
Supplement: Supplementary file 1 [file jcm-10-04063-s001.zip › jcm-1298522-supplementary.pdf]

SUPPLEMENTARY MATERIALS

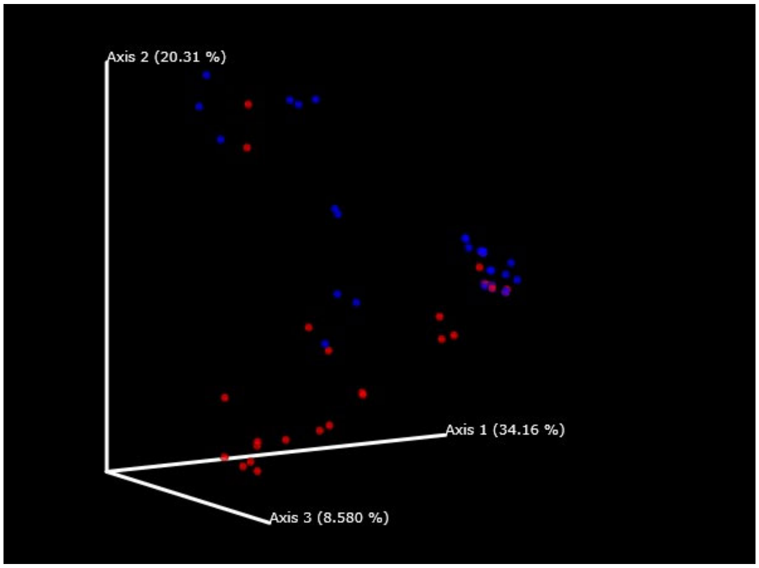

Figure S1. The unweighted UniFrac PCoA, based on the UniFrac distance matrix, which represents the study samples with different colors depending on the samples—vaginal (red) or endometrial (blue), QIIME2.

Figure S2

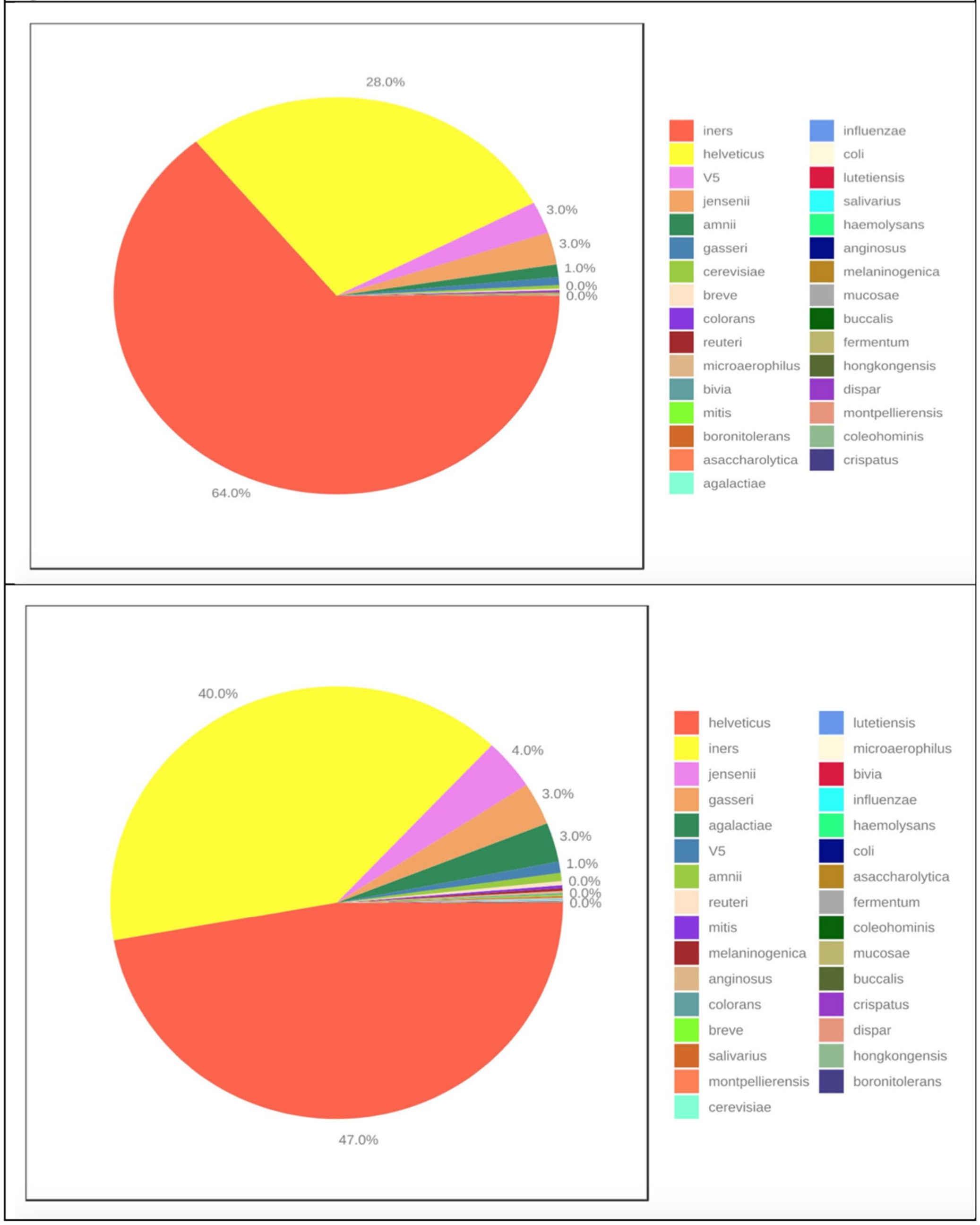

Figure S2. Pie chart of the relative frequency of the most abundant species for each sample type.

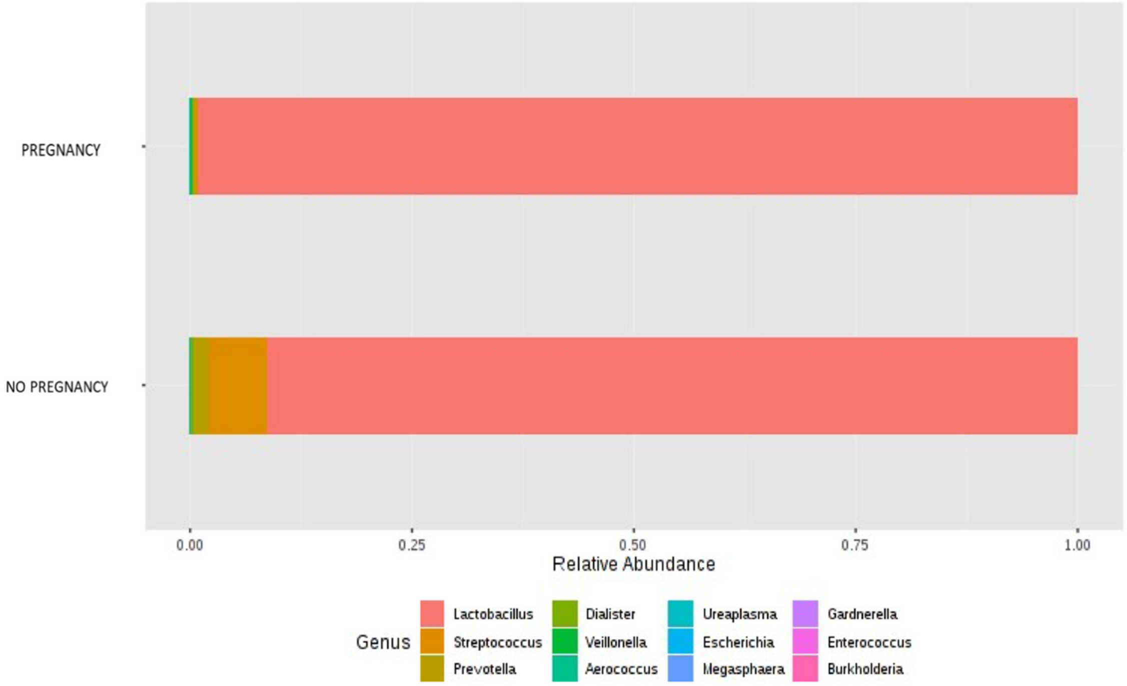

Figure S3. Bar chart of the relative frequency of the most abundant genera, grouped by gestation rate during the secretory phase of the cycle previous to frozen embryo transfer (days 18–22 of the cycle), MicrobiomeAnalyst MDP.

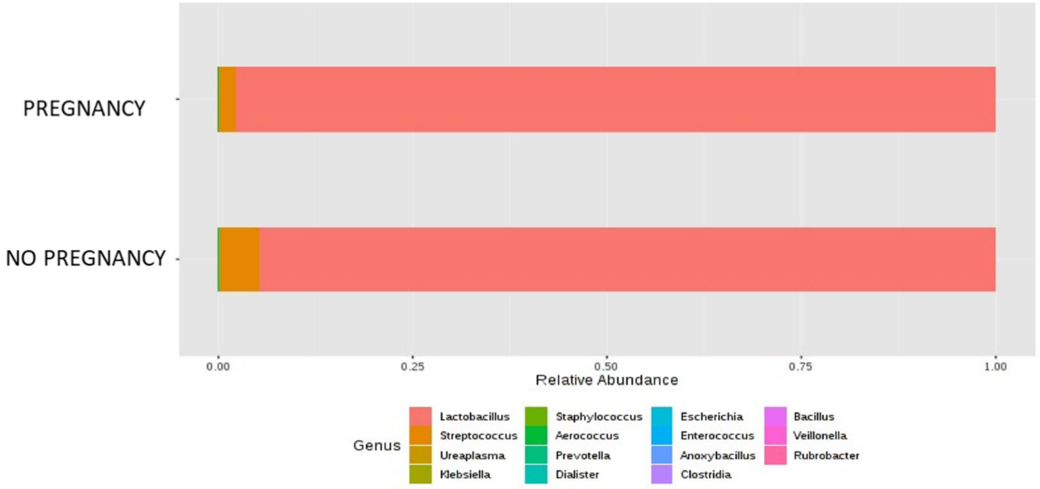

Figure S4. Bar chart of the relative frequency of the most abundant genera, grouped by gestation rate for the second timepoint, MicrobiomeAnalyst MDP.

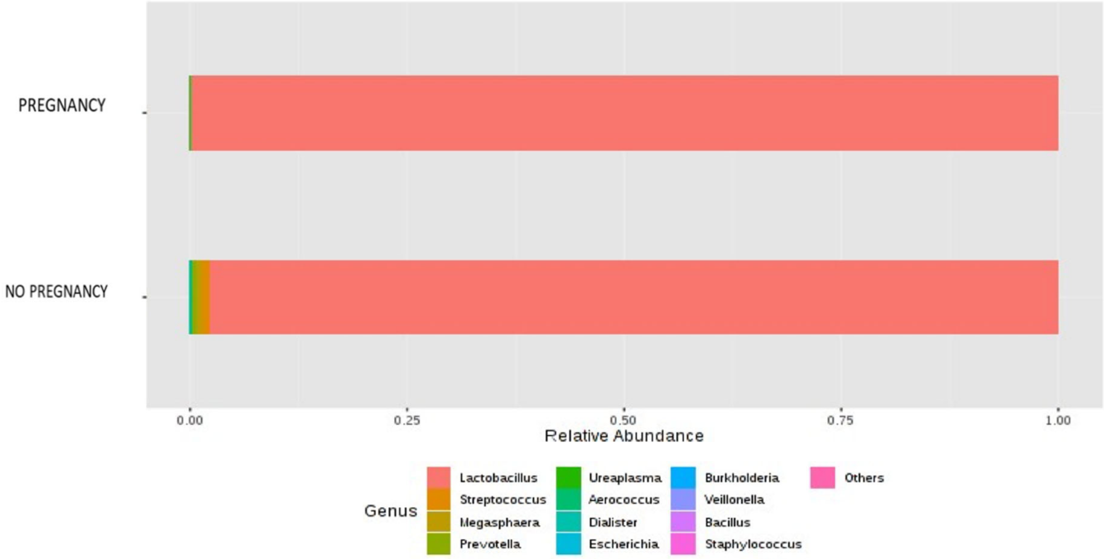

Figure S5. Bar chart of the relative frequency of the most abundant genera, grouped by gestation rate on the day of the pregnancy test, MicrobiomeAnalyst MDP.

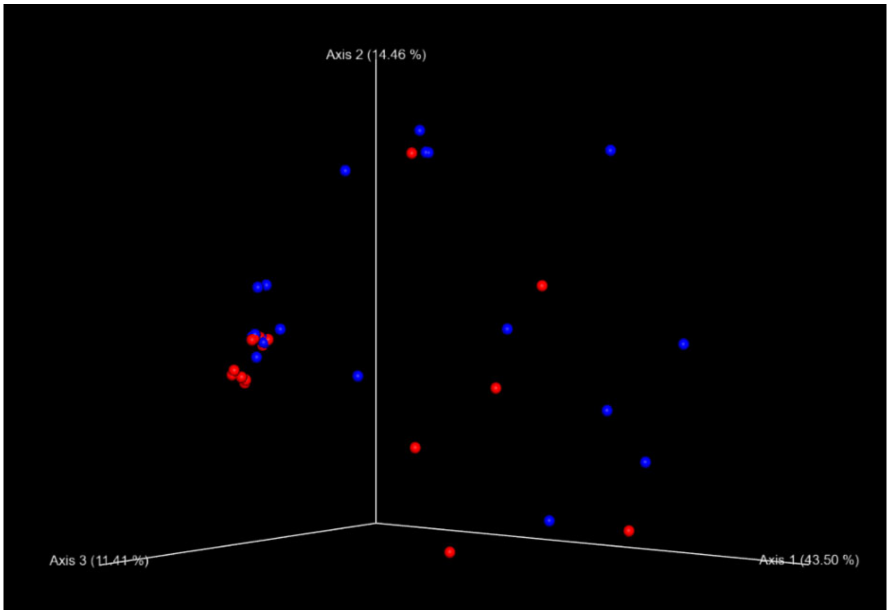

Figure S6. The unweighted UniFrac PCoA, based on the UniFrac distance matrix, which represents the study samples with different colors depending on the RIF diagnosis—vaginal (red) or endometrial (blue), QIIME2.

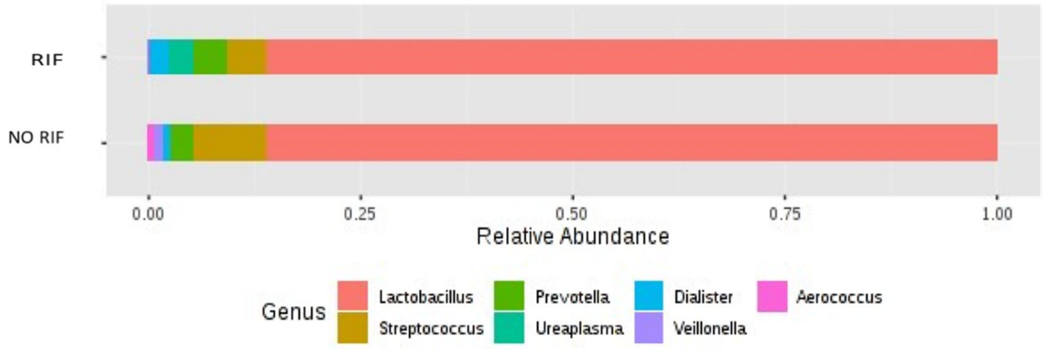

Figure S7. Bar chart of the relative frequency of the most abundant genera, grouped by RIF diagnosis, in the vaginal samples, MicrobiomeAnalyst MDP.

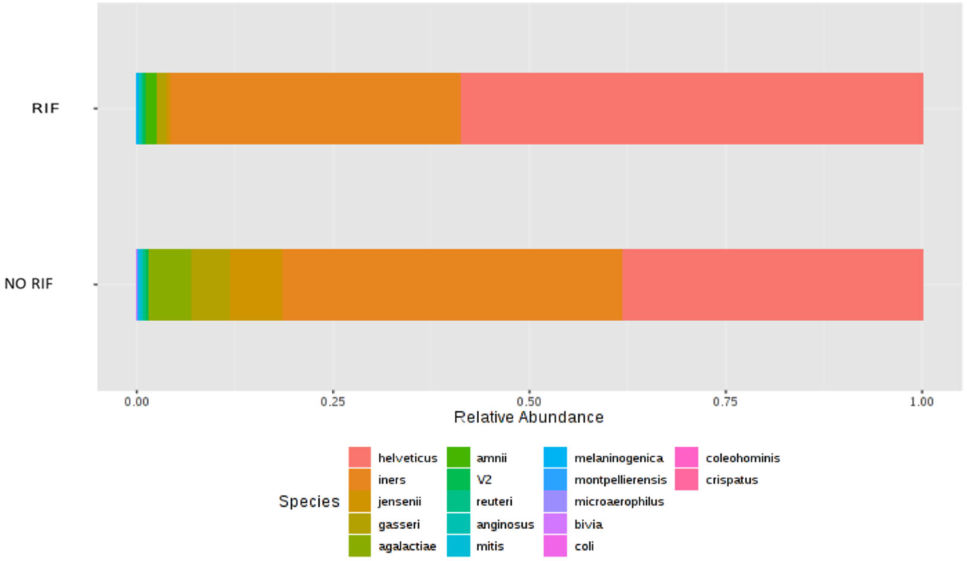

Figure S8. Bar chart of the relative frequency of the most abundant species, grouped by RIF diagnosis, in the vaginal samples, MicrobiomeAnalyst MDP.

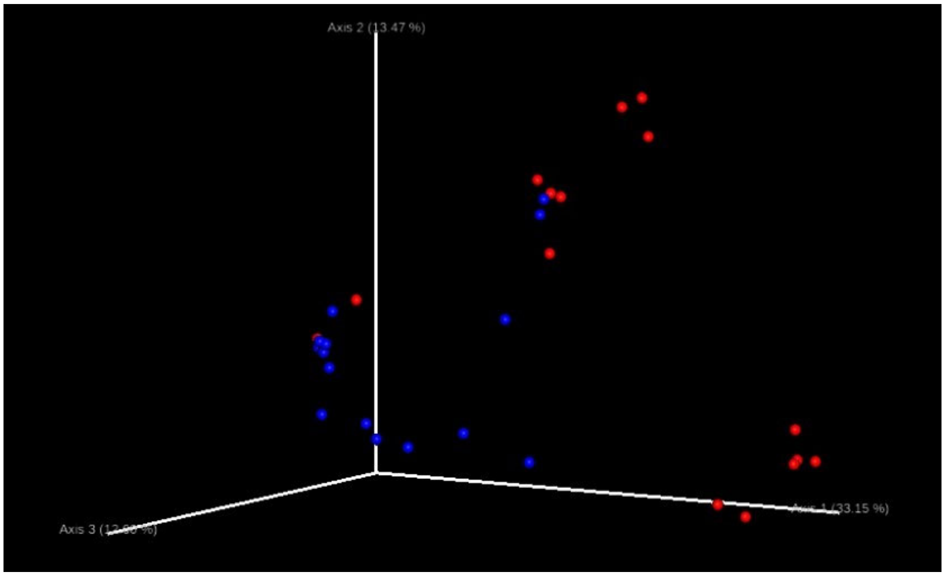

Figure S9. The unweighted UniFrac PCoA, which represents the women with and without RIF diagnosis using different colors—without RIF (red) or with RIF (blue), QIIME2.

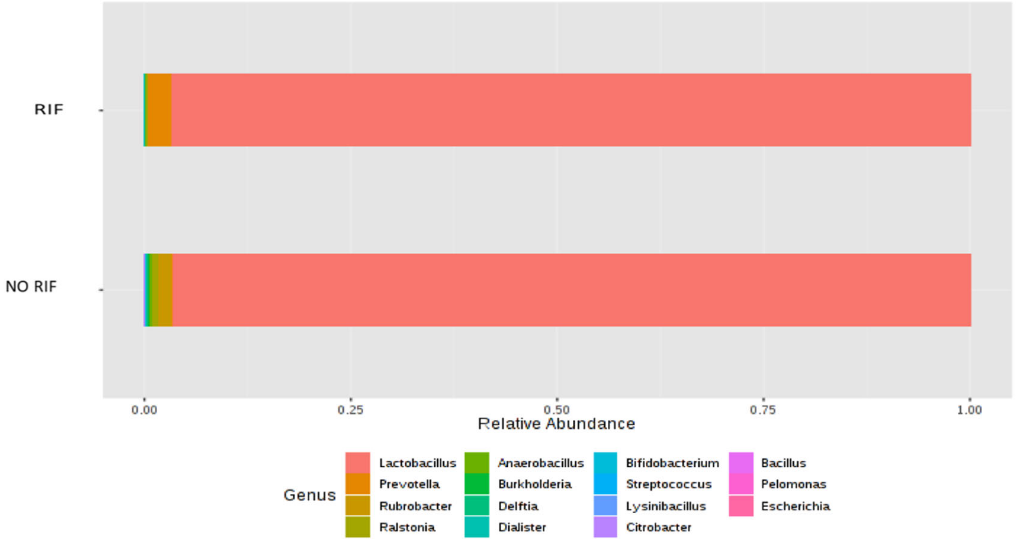

Figure S10. Bar chart of the relative frequency of the most abundant genera, grouped by RIF diagnosis, MicrobiomeAnalyst MDP.

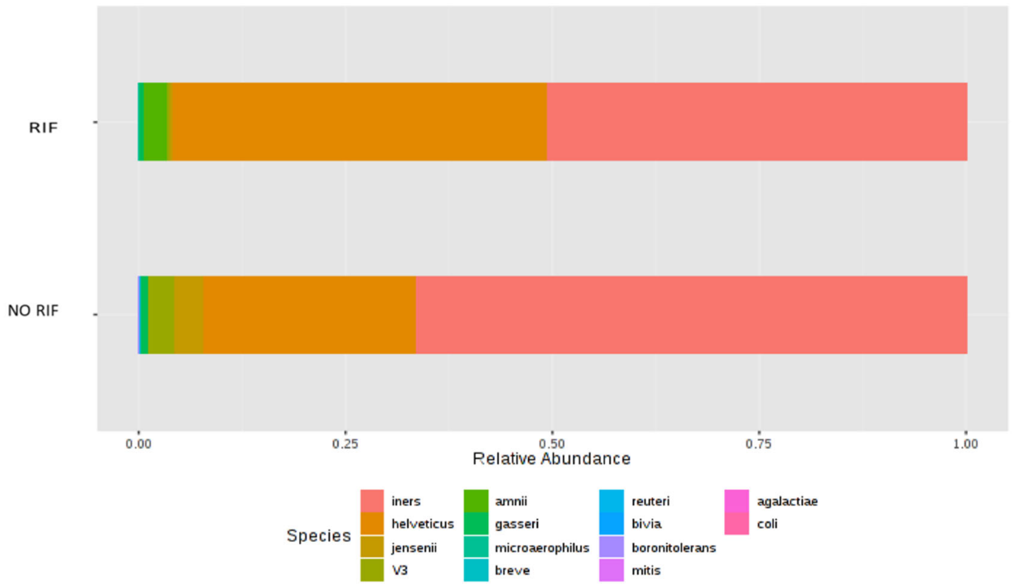

Figure S11. Bar chart of the relative frequency of the most abundant species, grouped by RIF diagnosis, MicrobiomeAnalyst MDP (*L. iners, L. helveticus, L. jensenii, L. gasseri, S. amnii*; V3 represents unidentified species).
